# Harmonised neuropsychological assessment for Vascular Cognitive Disorders: a Delphi consensus study

**DOI:** 10.1002/alz.086300

**Published:** 2025-01-03

**Authors:** Adam C. Bentvelzen, Nicole A. Kochan, Perminder S. Sachdev, VasCog‐2 Neuropsychological Consortium

**Affiliations:** ^1^ Centre for Healthy Brain Ageing (CHeBA), UNSW Sydney, Sydney, NSW Australia; ^2^ Centre for Healthy Brain Ageing (CHeBA), University of New South Wales, UNSW Sydney, NSW Australia; ^3^ Neuropsychiatric Institute, Prince of Wales Hospital, Randwick, NSW Australia

## Abstract

**Background:**

Harmonization of neuropsychological assessment for vascular cognitive disorders (VCD) is important for ensuring the highest standards for diagnostic and post‐diagnostic care. A battery jointly proposed by the NINDS‐CSN has received much support. Considering significant developments in the field, and an urgent need for consensus on remote and computerised assessment methods, an international expert group was commissioned to develop an updated harmonized battery and associated assessment guidelines for VCD using the Delphi process.

**Methods:**

A modified Delphi consensus method was used, involving an iterative, multi‐staged series of structured surveys with feedback of anonymized responses from experts in the neuropsychological assessment of vascular cognitive disorders. Three rounds were planned, with the possibility of a fourth round, if required to reach consensus. Literature reviews on harmonized neuropsychological assessment were conducted by a team of researchers, which informed the first structured questionnaire. Consensus was sought on the cognitive domains and subdomains that should be assessed, on specific tests per domain, and for additional guidelines including on non‐traditional assessment methods and cultural‐linguistic considerations. Consensus was defined as agreement or disagreement on any statement of ≥ 75%, near consensus as 66 – 75%, and <66% as non‐consensus. Statements that reached consensus were removed from subsequent rounds, as were most that had non‐consensus, with some being reworded. A virtual meeting was held for experts to discuss contentious issues and advise on the process.

**Result:**

Forty‐four experts in neuropsychological assessment from a range of international regions consented to participate, and 31 completed the Round 1 survey. The final survey is being completed, then the assessment battery and additional guidelines will be finalized to be approved formally by all participants.

**Conclusion:**

The harmonized neuropsychological assessment standards could be adopted internationally and complement the NINDS‐CSN battery, thereby further facilitating consistent neuropsychological assessment of VCD between clinicians and researchers.

